# Tolerability of Ziprasidone Use in Children and Adolescents: A Prisma Model: Systematic Review and Meta-Analysis

**DOI:** 10.1192/j.eurpsy.2022.383

**Published:** 2022-09-01

**Authors:** J. Jay, A. Sareen, N. Hassan, N. Dumlao, K. Jose, I. Haza, A. Wadhwa, S. Gunturu

**Affiliations:** 1 BronxCare Health System, Psychiatry, Bronx, United States of America; 2 University of Alabama, Psychiatry, Birmingham, United States of America

**Keywords:** metaanalysis, Ziprasidone, psychopharmacology

## Abstract

**Introduction:**

Studies have demonstrated that Ziprasidone use may be beneficial in children. Determining its potential risks and benefits when used in children is therefore important.

**Objectives:**

To examine the tolerability of Ziprasidone, an atypical antipsychotic, in children and adolescents.

**Methods:**

We conducted a literature search of open label or randomized control trials that report on Ziprasidone use in children on three databases: Embase, PsychInfo and PubMed using the PRISMA guidelines of Systematic review and Meta-analysis. Out of 1690 articles, 11 studies met inclusion criteria. Outcome measures included adverse effects such as weight gain, increase in BMI, QTc prolongation, changes in metabolic parameters, sedation, and dizziness. We conducted a random effects meta-analysis and meta-regression of potential moderators. Publication bias was assessed with funnel plots.

**Results:**

Data from Eleven studies was meta-analyzed (Total n= 474, mean age=12.87 years, male= 68..37%) that reported the use of Ziprasidone in children and adolescents with Psychosis, Bipolar, Autism spectrum disorders and Tourettes syndrome. Mean Ziprasidone dose = 84.40 mg and mean study duration = 2.85 months). We found that Ziprasidone was not found to cause any significant weight gain (1.72, p>0.05) or change in BMI (0.58 , p>0.05). QTc prolongation was found to be significant (11.9 , p<0.05). Most common side effects were sedation (42.44%), Nausea(19.32%), Headache (22.92%), fatigue (16.67%) and Dizziness (16.96 %).

**Conclusions:**

Results demonstrate that Ziprasidone does not cause significant weight gain, however QTc prolongation and sedation were found to be significant side effects of Ziprasidone use. Therefore, baseline EKG and thorough history must be obtained before prescribing Ziprasidone in children and adolescents.

**Disclosure:**

No significant relationships.

